# Supplementary material for: From sequence to enzyme mechanism using multi-label machine learning
Source: BMC Bioinformatics. 2014 May 19;15:150. doi: 10.1186/1471-2105-15-150 (PMC4229970; doi:10.1186/1471-2105-15-150)
Supplement: Additional file 2 — Java code of ml2db. Additional file ml2db_code.tar.gz contains the Java source code to run the multi-label machine learning experiments and save the results to database. The code’s Javadoc is included. [file 1471-2105-15-150-S2.zip › additional file 2/ml2db/utils/doc/uk/ac/ed/inf/utils/database/class-use/TableRow.html]

Uses of Class uk.ac.ed.inf.utils.database.TableRow


---


|  |  |  |
| --- | --- | --- |
| **Overview** \| **Package** \| **Class** \| **Use** \| **Tree** \| **Deprecated** \| **Index** \| **Help** | |  |
| PREV   NEXT | **FRAMES**    **NO FRAMES**     **All Classes** |  |


---


## **Uses of Class uk.ac.ed.inf.utils.database.TableRow**

| Packages that use TableRow | |
| --- | --- |
| **test.database** |  |
| **uk.ac.ed.inf.utils.database** |  |

Uses of TableRow in test.database

| Methods in test.database that return TableRow | |
| --- | --- |
| `static TableRow` | `TableRowTest.getRowKo()` |
| `static TableRow` | `TableRowTest.getRowOk()` |

Uses of TableRow in uk.ac.ed.inf.utils.database

| Methods in uk.ac.ed.inf.utils.database that return TableRow | |
| --- | --- |
| `TableRow` | `Table.createRow(java.lang.Object[] values, TableManager manager)` |
| `TableRow` | `TableRowSaver.fromValuesToRow(java.lang.Object[] values)`             Only works if the array contains all the values needed for the row and compatible with the row columns in the table object! TODO implement checks |
| `TableRow` | `TableRowSaver.getCurrentRow()`             Get the last table row saved or being saved |
| `TableRow` | `TableReader.getRowValues(java.sql.ResultSet resultSet, int rowNumber)`             Get the nth row of a `ResultSet` |
| `TableRow` | `TableReader.getTableRowWhereEqual(java.lang.String column, java.lang.String value)`             Gets the \*first\* table row where the given column has exactly the given value |
| `TableRow` | `TableReader.getTableRowWhereLike(java.lang.String column, java.lang.String value)`             Gets the \*first\* table row where the given column has a value similar to the given value |

| Methods in uk.ac.ed.inf.utils.database with parameters of type TableRow | |
| --- | --- |
| `java.lang.String` | `TableRowSaver.dataToString(TableRow row, java.lang.String filePath)` |
| `java.lang.String` | `TableWriter.getInsertSql(TableRow row)`             Returns the sql "insert into" statement for the row |
| `java.lang.Integer` | `TableWriter.insertReturnsKey(TableRow row)`             Executes an sql 'insert into' operation. |
| `int` | `TableRowSaver.saveAndPrintRow(TableRow row, java.lang.String filePath)` |
| `int` | `TableRowSaver.saveRowToTable(TableRow row)` |
| `java.lang.Integer` | `TableWriter.writeRowToTable(TableRow row)`             Insert the row values into the appropriate database table |

Constructors in uk.ac.ed.inf.utils.database with parameters of type TableRow

`TableRowSaver(TableRow tableRow)`

---


|  |  |  |
| --- | --- | --- |
| **Overview** \| **Package** \| **Class** \| **Use** \| **Tree** \| **Deprecated** \| **Index** \| **Help** | |  |
| PREV   NEXT | **FRAMES**    **NO FRAMES**     **All Classes** |  |


---
